# Glucocorticoids influence on rat hematological parameters and catalase activity

**DOI:** 10.3389/fphar.2024.1367350

**Published:** 2024-11-13

**Authors:** Safija Herenda, Ivana Carev, Denis Haskovic, Sabina Prevljak, Sara Causevic, Edhem Haskovic

**Affiliations:** ^1^ Department of Chemistry, Faculty of Science, University of Sarajevo, Sarajevo, Bosnia and Herzegovina; ^2^ Faculty of Chemistry and Technology, University of Split, Croatia, Croatia; ^3^ Faculty of Science, University of Split, Split, Croatia; ^4^ Organizational Unit Clinical pathology, Cytology and Human Genetics, Clinical Center of the University of Sarajevo, Sarajevo, Bosnia and Herzegovina; ^5^ Organizational Unit Clinic for Radiology, Clinical Center of the University of Sarajevo, Sarajevo, Bosnia and Herzegovina; ^6^ Department of Public Health Sciences, Stockholm University, Stockholm, Sweden; ^7^ Department of Biology, Faculty of Science, University of Sarajevo, Sarajevo, Bosnia and Herzegovina

**Keywords:** enzymes, drug research, biosensors, glucocorticoids, rat, kinetics

## Abstract

In this study, the impact of glucocorticoid, betamethasone dipropionate on enzyme activity *in vitro* and its effects on hematological parameters *in vivo* was investigated. The immobilized catalase, crucial for cell oxidative stress response via hydrogen peroxide reduction, exhibited a robust electrocatalytic response, maintaining its biological activity. The *in vitro* inhibition kinetics of catalase, as determined by electrocatalytic methods and expressed using Lineweaver-Burke diagrams, revealed an uncompetitive type of inhibition with altered Imax and Km in the presence of a range of betamethasone dipropionate concentrations. The *in vivo* experiments conducted on *Rattus norvegicus* demonstrated significant alterations in hematological parameters following betamethasone dipropionate administration. These changes included a decrease in erythrocyte count, an increase in hemoglobin, a reduction in mean corpuscular volume (MCV), and an elevation in mean corpuscular hemoglobin (MCH) and mean corpuscular hemoglobin concentration (MCHC). Notably, the leukocyte counts substantially increased. The observed hematological shifts suggest an impact of betamethasone dipropionate on the hematopoietic system, reinforcing the need for cautious corticosteroid administration. The findings underline the necessity for judicious corticosteroid treatment, acknowledging both enzymatic and systemic repercussions.

## Graphical Abstract

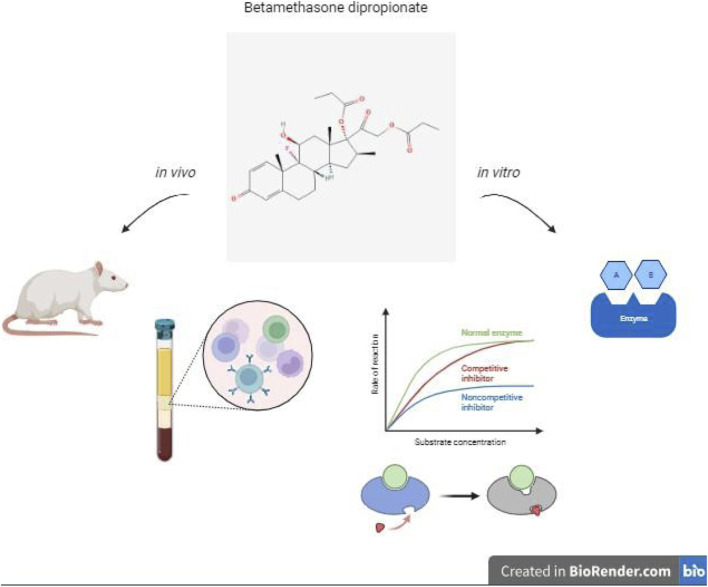

## 1 Introduction

Drugs interact with proteins and other macromolecules in body fluids and tissues, and such binding interactions affect the pharmacokinetics of the drugs (Deutsch et al., 2013). A significant part of the drug in the body can reversibly interact with proteins and many cells in a non-specific way. Such nonspecific binding is the primary determinant of drug distribution in the body and plays an important role in pharmacokinetics (Ayyar et al., 2019). Glucocorticoids are the primary stress hormones that regulate various physiological processes, and their action is mediated by the glucocorticoid receptor (GR). Glucocorticoid receptors are present in nearly every cell of vertebrate animals. However, there is notable variation in how different tissues respond to glucocorticoids and their sensitivity to these hormones. Corticosteroids as enzyme inhibitors are widely used as pharmacological agents to treat pneumonia, asthma, and immune/rheumatological disease. They possess both anti-inflammatory and immunosuppressive properties (Ramamoorthy and Cidlowski, 2016).

Betamethasone dipropionate (C28H37FO7) is a synthetic glucocorticoid, a derivative of prenisolone, 16β-methyl-prednisolone, possessing immunosuppressive and anti-inflammatory properties. Betamethasone dipropionate is a corticosteroid commonly used to treat various inflammatory and autoimmune disorders (Haskovic et al., 2022). Corticosteroids are metabolized in the liver by cytochrome P450 3A4 (CYP450) through enzymatic transformations that reduce their physiological activity and increase solubility in water to increase their excretion in the urine. The long-term use of corticosteroids can lead to various side effects, including inhibiting enzymatic activity (McKay and Cidlowski, 2024).

Catalase is an enzyme that plays a crucial role in protecting cells from oxidative damage by catalyzing the decomposition of hydrogen peroxide into water and oxygen. Inhibition of catalase activity can lead to the accumulation of reactive oxygen species (ROS) and oxidative stress, contributing to the development of various diseases. Catalase is a chemical protein that belongs to the group of oxidoreductases with ferriprotoporphyrin-IX in the redox center. It catalyzes the disproportion of hydrogen peroxide into oxygen and water without the formation of free radicals (Kodydková et al., 2014). Understanding the impact of betamethasone dipropionate on catalase activity is essential for developing safer and more effective treatments for inflammatory and autoimmune disorders (Brazzini and Pimpinelli, 2002). Many methods of monitoring drug binding in the body have been developed. Those *in vivo* methods measure the drug uptake into tissues after the balance state between plasma and target tissue is established (Khalafallah and Jusko, 1984). Most drugs today used in clinical treatments, base their mode of action on enzyme inhibition (Copeland, 2013). Direct electrochemistry methods of enzyme activity can provide an crucial information on the mechanism of enzymatic activity and electron transfer in biological systems (Seckl, 2004).

This study aims to investigate the influence of corticosteroids betamethasone dipropionate on hematological parameters *in vivo* and inhibitory properties on catalase activity *in vitro*. *In vivo* tests were conducted on rats *Rattus norvegicus* (Berkenhout 1769), where hematological parameters were examined: leukocyte count, erythrocyte count, hemoglobin concentration, hematocrit, and hematological indexes (MCV, MCH and MCHC). By employing an electrochemical approach using cyclic voltammetry and chronoamperometry methods, the research seeks to elucidate the potential inhibitory effects of corticosteroid betamethasone dipropionate on the enzymatic activity of catalase to provide additional information about the mechanism of inhibition of this enzyme. Understanding the impact of this drug on enzyme kinetics holds significant promise for advancing our knowledge of their mechanisms of action. It may contribute to developing novel therapeutic strategies for conditions characterized by inflammation and autoimmune responses.

The hypothesis of our work is that the corticosteroid, specifically betamethasone dipropionate, will exert inhibitory effects on the enzyme catalase and influence hematopoietic parameters.

## 2 Results and discussion

The objective of this study was to examine the impact of corticosteroids betamethasone dipropionate on hematological parameters in living organisms and its ability to inhibit catalase activity in laboratory conditions.

For the purpose of examining the effect of betamethasone dipropionate on catalase activity an electrochemical approach using cyclic voltammetry and chronoamperometry methods were used.

In an enzyme sensor, signals that are caused by enzymatic reactions are converted into an electrical signal by GC electrode (D’Souza, 2001). The immobilized enzyme exhibits good sensitivity, selectivity, as well as good response time. Many types of oxidoreductases catalytically transport electrons to the corresponding electron acceptors. Redox responses can be obtained when stimulated by various enzyme substrates such as H_2_O_2_, NADH, ethanol and others. The kinetic parameters obtained by analysis based on the Michaelis-Menten equation may include the effect of mass transfer resistance through the outer membrane of the enzyme, but the effect was estimated as small (Ikeda et al., 1996).

**FIGURE 1:**
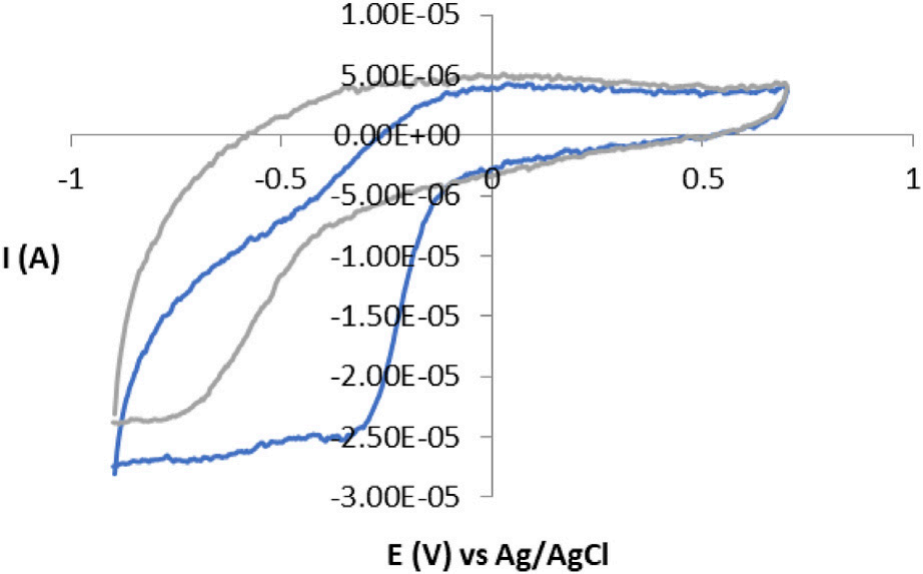
Cyclic voltammogram without enzyme immobilization (gray) and with immobilized enzyme (blue), at a scanning speed of 50 mV/s.

Detailed mechanistic research into electron transfer between electrodes and enzymes can be very complex, especially if the enzyme reaction is multiple, such as the reaction of the enzyme peroxidase and hydrogen peroxide (H_2_O_2_). In our work, the reaction of catalase and hydrogen peroxide took place according to a simple mechanism and with high efficiency. The enzyme is first oxidized to a high-valence intermediate of iron, known as compound I (Cpd I) which, unlike other hydroperoxides, returns to rest by further reaction with H_2_O_2_. The ideal cyclic voltammogram represents symmetric spades of oxidation and reduction with formal potential (E0) on the surface of the redox reaction (Murray, 1980). The cyclic voltammogram of the GC electrode without and with immobilized catalase in the phosphate buffer pH = 7 at 50 mV/s is presented in Figure 1. For the immobilization of catalase on the GC electrode, as well as for other enzymes, polymers are used, as well as other materials, and their efficiency depends on the thermodynamics of the redox reaction in the biosensor, the kinetics of electron transfer between the biosensor and the electrode as well as the transfer of charge within the film on the electrode (biosensor) (Mirkin, 2003). Films thicker than the electroactive enzyme monolayer can often provide a higher enzyme load per unit area of the electrode resulting in larger current peaks. Charge transfer through these films may involve physical diffusion of enzymes and/or electron self-exchange reactions between the redox centers of the enzyme (Blauch and Saveant, 1992). The immobilization approach offers a direct avenue for investigating the electrochemical properties of redox enzymes and enzyme catalysis through direct voltammetry without the need for mediators. A particularly valuable analytical strategy involves plotting reduction and oxidation peak potentials against the logarithm of scan rate across a broad range. This approach facilitates mechanistic studies, allowing exploration of enzyme behavior in the absence and presence of the substrate.

**FIGURE 2:**
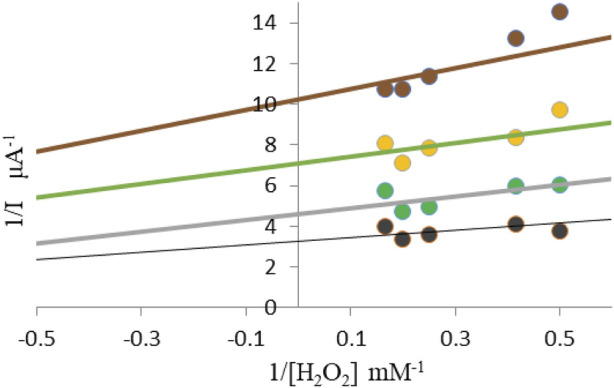
Lineweaver–Burke diagram for determination of I_max_ and K_m_ without the presence and presence of different concentrations of corticosteroids black line 0 mM; grey line 1,013 mM; green line 2,026 mM; brown line 3,039 mM.

Chronoamperometric method monitored catalytic reduction current in the presence of different concentrations of hydrogen peroxide (1.6–6 mM) in saline. In the Lineweaver-Burke diagram, the type of inhibition and maximum current values (I_max_) and the Michaelis-Menten constant (K_m_) are determined without and in the presence of inhibitors (Figure 2).

The Lineweaver-Burke diagram shown in Figure 2 shows that uncompetitive type of inhibition is present in the enzymatic reactions performed. The obtained values of kinetic constants Imax and Km change with the addition of different concentrations of inhibitors Imax (0.14 μA; 0.21 μA; 0.30 μA), while the values of Km (0.47 mM; 0.62 mM; 0.55 mM). Inhibitors of this modality require the pre-formation of ES complexes for binding and inhibition. The characteristic of this type of inhibition is that the inhibitor reversibly binds to the enzyme-substrate complex, resulting in an inactive enzyme-substrate-inhibitor complex that leads to the formation of the product. The binding of corticosteroids to the ES complex occurs according to the following mechanism (Marangoni, 2002):
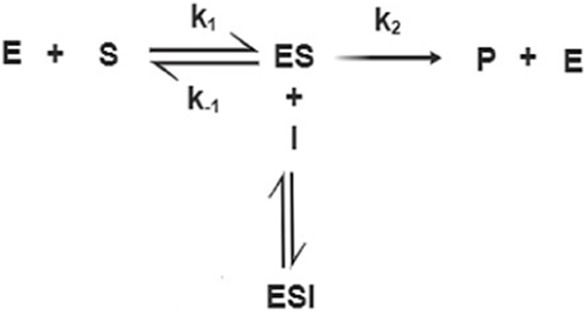


In this case, the Lineweaver-Burk diagram takes on another form:

$$\frac{1}{v_{0}}=\frac{K_{M}}{V_{\max}}\frac{1}{\left[S\right]}+\frac{1}{V_{\max}}\left(1+\frac{\left[I\right]}{K_{I}}\right)$$

An intermediate product of betamethasone dipropionate occurs when the transfer of electrons occurs from the substrate to the double bond of glucocorticoids. This enolate intermediate is stabilized by interacting with an acid group within the enzyme-active site. Then, in the reaction, proton donation from the active site to enolate carbon occurs, forming the product of the reaction (Copeland, 2013). The affinity of accumulating inhibitors is highest at saturated substrate concentrations.

Depending on the physiological conditions this characteristic of high substrate concentrations to overcome inhibitors may offer some clinical advantage to these inhibition modalities. It cannot be known which inhibition modality will be most effective in *vivo* research other than empirical studies, therefore the diversity of inhibition modalities should always be the goal, at least at an early stage of the drug discovery program (Copeland, 2013). The authors of Copeland and Anderson examined the inhibition of epristeride to the enzyme steroid 5a-reductase, where they also proved uncompetitive binding. This enzyme binds the cofactor NADPH at its active site and then binds the male hormone testosterone to form the ternary enzyme complex-NADPH-testosterone. The intermediate testosterone enolate occurs when there is a stereospecific transfer of hydrides from NADPH to the *β*-carbon double bond of testosterone. This enolate intermediate is stabilized by interacting with an acid group within the enzyme’s active site. Eventually, a proton donation occurs from the base of the active site to the enolate α-carbon forming a product of the dihydrotestosterone reaction (DHT), with the release of NADP + to complete the reaction cycle (Copeland, 2013). In our previous work we have demonstrated the same, uncompetitive type of inhibition of the range of concentrations of betamethasone dipropionate on catalase, using spectrophotometry method. The advantage of our work in this study is the use of electrochemical methods that can help us facilitate mechanistic studies, allowing exploration of enzyme behavior in the absence and presence of the substrate. In instances where enzyme thin-film voltammetry is reversible at low scan rates but exhibits electron-transfer kinetic limitations at high scan rates, the resulting plot illustrates nearly equal and constant oxidation-reduction peak potentials at low scan rates, transitioning symmetrically in opposite directions at higher scan rates. Additionally, this study has highlighted the highest affinity of accumulating inhibitors at saturated substrate concentrations, suggesting potential clinical advantages in overcoming uncompetitive inhibition. Smajdor, et al. examined the electrochemical high sensitive voltammetric determination of betamethasone on an amalgam film electrode.

The new procedure was successfully applied for betamethasone determination in different types of drug samples with very good recovery, calculated in the range from 95% to 105% (Smajdor et al., 2018). New study investigates the corrosion inhibition of copper in a sulfuric acid solution using varying concentrations of Expired Betamethasone Drug, employing weight loss and Experimental Design methods.

Results show that inhibition efficiency increases with higher inhibitor concentrations but decreases with rising temperature. Thermodynamic analyses elucidate adsorption and activation processes, revealing that the adsorption of Expired Betamethasone Drug on copper surfaces is characterized as endothermic and spontaneous, aligning well with the Langmuir and Frumkin adsorption isotherms (Attar and Benchadli, 2024).

For the purpose of examining the effect of betamethasone dipropionate on living organisms, we have tested set of doses on animal model of laboratory rat, followed by measurement of hematological parameters after heart punction. The sex and body weight of the experimental groups of rats, belonging to the species *R. norvegicus* (Berkenhout, 1769), were determined using morphometric parameters. The hematological parameters included total leukocyte count, erythrocyte count, hemoglobin concentration, hematocrit, and hematological indices such as CVD, MCH, and MCHC. Once the blood samples were collected and analyzed, the were statistically processed using ANOVA work package and *t*-test. Analysis of variance (ANOVA) is a statistical test used to assess the difference between the means of more than two groups. *t*-test refers to testing the statistical significance of the difference between two arithmetic means. The resulting difference between both arithmetic means is divided by the standard error of that difference. Analysis and processing of results were done by the method of descriptive and analytical statistics using Microsoft Excel 2013, PAST (Paleontological Statistics) 3.25 and Minitab 19. The results are presented in Table 1.

**TABLE 1:** Results of hematological parameters of *Rattus norvegicus*.

Hematological parameters	Control group	Experimental group [betamethasone dipropionate] 0.3 mg/kg	Significance
Leukocyte (x 109/L of blood)	6.01 ± 0.47	9.00 ± 0.87	*p* < 0.05 t = 0.00
Erythrocytes (x 1,012/L of blood)	7.87 ± 0.44	7.30 ± 0.14	*p* < 0.05 t = 0.02
Hemoglobin (g/dL)	90.27 ± 53.64	203.22 ± 24.23	*p* < 0.05 t = 0.003
Hematocrit (L/L)	0.498 ± 0.08	0.239 ± 0.066	*p* < 0.05 t = 0.00
CVD (fL)	63.14 ± 8.51	32.74 ± 9.27	*p* < 0.05 t = 0.00
MCH (pg)	11.26 ± 6.07	27.83 ± 3.30	*p* < 0.05 t = 0.00
MCHC (g/dL)	173.87 ± 70.64	932.58 ± 393.35	*p* ≤ 0.05 t = 0.05

The circulatory system in every animal has a vital function in the transportation of plasma proteins and other components that are involved in metabolic activities. Ingesting a toxic component results in the distribution of this substance throughout the body via the circulatory system. Consequently, a rise in the concentration of this substance in the body would cause abnormalities in the affected animals. The application of betamethasone dipropionate to rats *R. norvegicus* led to a change in a list of hematologic parameters. (Table 1).

In our experiment, the results obtained from the studied hematological parameters show that the applied dose of betamethasone dipropionate in the experimental group leads to a decrease in the number of erythrocytes, causes an increase in hemoglobin value, reduces the value of CVD, and increases the value of MCH and MCHC and these differences are statistically significant. Also, administered doses of betamethasone dipropionate in individuals of the experimental group lead to an increase in the number of leukocytes compared to the value in the control group, and this difference is also statistically significant (*p* < 0.05). The administered dose of betamethasone dipropionate leads to a decrease in hematocrit values in individuals of the experimental group, which is also reflected in the values of MCV and MCHC. However, the same dose of betamethasone dipropionate in all ten (Seckl, 2004) individuals of the experimental group leads to a decrease in hematocrit values, and consequently leads to a change in the values of the hematological indexes MCV and MCHC. The change in these parameters was determined experimentally, which shows that betamethasone dipropionate in some way affects the organism, especially the hematopoietic system Research by authors in animal models shows that prenatal glucocorticoids cause a number of serious side effects depending on the dose, gestational age of the fetus and the species used.

Steroid treatment during the third trimester has been shown to cause: growth retardation, decrease in cerebellar DNA content, decreased brain growth, hypomyelination, increased sensitivity of cerebellum neurons to oxidative cell death, and improvement in the maturation state of neural cells already present during treatment (Velazquez and Romano, 1987; LaBorde et al., 1992; Huang et al., 1999).

In our previous research of betamethasone dipropionate on rat hematological parameters we used different approach, experimental design, and presentation of results (Haskovic et al., 2022). In current study we focus on a single experimental group with detailed observations of the effects of betamethasone dipropionate effects on the hematopoietic system, especially in the context of prenatal glucocorticoid exposure in animal models. In the work of Haskovic, et al., two doses of betamethasone 0.2 mg/kg and 0.4 mg/kg were examined for hematological parameters, the samples were taken for 5 days and all females in that work were fed high-calorie food. Comparing the results, we came to the conclusion that this particular food was the cause of higher values of leukocytes at the dose of 0.3 mg/kg (9.00 ± 0.87), while the values of leukocytes at the dose of 0.2 mg/kg (4.93 ± 0.25) and at the dose of 0.4 mg/kg (6.51 ± 0.23).

The therapeutic effect of corticosteroids is based on their action on the synthesis of lipocortin and vasocortin, inhibiting the formation of edema and the enzyme A2 phospholipase. By inhibiting this enzyme, membrane phospholipids cannot be converted into arachidonic acid. Therefore, the synthesis of prostaglandins and prostacyclines as well as the synthesis of leukotrienes synthesized from arachidonic acid is blocked (Manchikanti, 2002). Nakanishi, et al. point out that pulp fibroblasts and macrophages may participate in the production of prostaglandins through the expression of cyclooxygenase-2 in inflammation of the pulp, and both may be involved in the pathogenesis of pulpitis. Therefore, the use of anti-inflammatory agent inhibits edema, vasodilation and chemotactic effect on leukocytes (Nakanishi et al., 2001).

The results obtained in the study of the influence of betamethasone dipropionate on hematological parameters in women in late pregnancy are similar to our study. This study showed that the administration of betamethasone dipropionate in women in late pregnancy caused transient neutrophilia and leukocytosis. The extent and duration of these changes may be useful in conducting clinical treatment for women receiving betamethasone dipropionate and who are at risk of perinatal infection. The differences between steroid regimens emphasized the need to evaluate the hematological effects of each regimen separately (Wallace et al., 1998).

## 3 Conclusion

In this study, the primary goal was to investigate the impact of the corticosteroid betamethasone dipropionate on both hematological parameters *in vivo* and its inhibitory properties on catalase activity *in vitro*. Recognizing the critical role of drug-protein interactions in pharmacokinetics, the research focused on glucocorticoids and we hypothesized that betamethasone dipropionate would inhibit catalase activity and influence hematopoietic parameters. Using an electrochemical approach with cyclic voltammetry and chronoamperometry methods, the study explored the potential inhibitory effects of betamethasone dipropionate on catalase enzymatic activity, *in vitro*. The *in vivo* component involved testing 0.3 mg/kg dose of betamethasone dipropionate on laboratory rats and assessing hematological parameters, including leukocyte count, erythrocyte count, hemoglobin concentration, hematocrit, and hematological indices. The results demonstrated significant alterations in these parameters, indicating the drug’s influence on the hematopoietic system. The electrochemical methods provided mechanistic insights into the inhibitory effects on catalase, showing an uncompetitive type of inhibition and highlighting the potential clinical advantages of such inhibition modalities. The study contributes valuable information to the understanding of betamethasone dipropionate’s impact on enzyme kinetics and hematological parameters, offering insights for developing novel therapeutic strategies for conditions involving inflammation and autoimmune responses. Comparisons with previous research underscore the importance of evaluating the hematological effects of corticosteroids independently for different regimens.

## 4 Materials and methods

For the purpose of this work the *in vivo* preclinical animal testing, where hematological parameters were examined: leukocyte count, erythrocyte count, hemoglobin concentration, hematocrit and hematological indexes (MCV, MCH and MCHC); and *in vitro* electrochemical methods of cyclic voltammetry and chronoamperometry were used for measurements of betamethasone dipropionate activity.

Chemicals: Enzyme catalase CAT (c100-50 MG) Sigma-Aldrich (Buchs, Switzerland); KH_2_PO_4_ and Na_2_HPO_4_, Fisher Chemical (Wien, Austria); hydrogen peroxide (H_2_O_2_) p. a. 30%, Sigma-Aldrich (Buchs, Switzerland); Nafion Sigma-Aldrich (Buchs, Switzerland); Betamethasone dipropionate, Bosnalijek, BiH.

Electrochemical methods: The instrument used for measurements is potentiostat/galvanostat PAR 263A with a classicthree-electrode system in which the saturated Ag/AgCl electrode was used as a reference electrode, the Pt-electrode as the counter electrode, and GC (glassy carbon) as the working electrode. An amperometric biosensor was formed to determine H_2_O_2_ by immobilizing an enzyme (CAT) to the surface of the GC electrode trapped in the Nafion layer described in the literature (Herenda et al., 2018; Ostojić et al., 2017). Cyclic voltammetry was used to examine immobilization, an enzymatic film on the surface of the electrode. All cyclic voltammetry tests were performed in saline phosphate buffer (pH = 7) and potential range from −1.0 to 1.0 V, at a scanning speed of 50 mV/s. Chronoamperometric technique has been used to determine kinetic parameters: the Michaelis-Menten constant (K_m_) and the maximum current value when the solution is saturated with a substrate (I_max_) equivalent to the maximum reaction rate (V_max_), as well as to determine the type of inhibition (Herenda et al., 2018). Chronoamperometric measurements were performed in the cell of 25 mL phosphate buffer at a constant potential of 0.9 V imposed on the working electrode, as well as at constant mixing of 400 rpm. The reaction was observed without the presence and with the presence of different concentrations of betamethasone dipropionate.

Preclinical testing on animals: studies were conducted on laboratory rats of the species *R. norvegicus*; strain Wistar (Berkenhout, 1769), bred in the vivarium of the Department of Biochemistry and Physiology, Department of Biology, University of Sarajevo, Bosnia and Herzegovina. Animal handling, care, and treatment were all required throughout the experimental phase. In Bosnia and Herzegovina, experiments involving animals are subject to the Law on the Protection and Welfare of Animals (Official Gazette of BiH, Nos. 25/2009 and 9/2018). The animals were also taken care of per the “Declaration on Animal Rights” (UNESCO, 1978) and “Universal Declaration on Animal Welfare” (WSPA, 2000). The animal study was approved by Faculty of Science, University of Sarajevo. The laboratory rat, *R. norvegicus*, belongs to the order Rodentia family Muridae They are characterized by short fur coats, and a long bare tail, rounded upright ears, a pointed muzzle with long vibrations and five fingers on each foot. Laboratory rats of Wistar strain are albino due to a mutation in the gene for the enzyme tyrosinase, have red eyes and poor vision. They can reach a length of up to 400 mm and a weight of 140–500 g (Haskovic et al., 2022).

For the experiment we have used 20 *R. norvegicus* individuals, 10 in control group and 10 individuals who were administered intraperitoneal (i.p.) betamethasone dipropionate at a concentration of 0.3 mg/kg body weight (Drugs.com, 2023). At the moment of administration, the rats were 3 months old, all individuals were bred in the same environmental conditions, and at the very beginning of the study individuals were determined body weight to administer the correct dose of betamethasone dipropionate. The heart puncture was performed 4 hours after the application of betamethasone dipropionate for the purpose of taking the blood for examining the effect of betamethasone dipropionate on the hematological parameters of rats. During heart puncture, syringes with EDTA (ethylenediaminetetraacetic acid) were used, from which blood was transferred to a test tube also from EDTA, which was used as an anticoagulation agent. The analysis of hematological parameters was done immediately after the completion of the heart puncture.

The determination of the number of leukocytes (WBC) was carried out by the standard method in the hemocytometer using Türk’s reagent. Differential blood counts were determined microscopically, by examination of peripheral blood smears that were stained by Pappenheim method (MS et al., 1990). The determination of the number of erythrocytes (RBC) was carried out by the standard method in the hemocytometer using Hayem’s reagent. The concentration of hemoglobin was determined spectphotometrically using Drabkin’s reagent. The method is based on the oxidation of hemoglobin and its derivatives other than sulfhemoglobin in the presence of potassium ferrichanide to methemoglobin which reacts with potassium cyanide to a stable compound cyanmethemoglobin which has a maximum absorption at 540 nm (Haskovic et al., 2022).

To determine hematocrit, we used the microhematocrit method, centrifuging blood for 5 minutes at 16,000 rpm and 139.18 RCF. Hematocrit (Hct) represents the ratio of blood elements to plasma obtained after centrifugation. Hematological indices the average volume of erythrocytes (MCV), the average amount of hemoglobin in one erythrocyte (MCH) and the average concentration of hemoglobin in a liter of erythrocytes (MCHC) were calculated mathematically based on the values of the total number of erythrocytes, hematocrit and hemoglobin.

## Data Availability

The raw data supporting the conclusions of this article will be made available by the authors, without undue reservation.
